# Characteristics of ScleroID highlighting musculoskeletal and internal organ implications in patients afflicted with systemic sclerosis

**DOI:** 10.1186/s13075-023-03063-1

**Published:** 2023-05-20

**Authors:** Gabriella Nagy, Rucsandra Dobrota, Mike Oliver Becker, Tünde Minier, Cecília Varjú, Gábor Kumánovics, Oliver Distler, László Czirják

**Affiliations:** 1 Department of Rheumatology and Immunology, University of Pécs, Medical School, Pécs, Hungary; 2 Department of Rheumatology, University Hospital Zurich, University of Zurich, Zurich, Switzerland

**Keywords:** Patient-reported outcome measure, Systemic sclerosis, Health-related quality of life, Hand function, Disease activity

## Abstract

**Background:**

Systemic sclerosis (SSc) is a multi-organ disease with impaired health-related quality of life (HRQoL). The EULAR SSc Impact of Disease (ScleroID) is a newly introduced SSc-specific patient-reported outcome to evaluate HRQoL in SSc.

**Objective:**

To investigate the correlation between the ScleroID and the involvement of organ systems as well as disease activity/damage in a SSc cohort from a large tertiary care centre.

**Patients and methods:**

The ScleroID and clinical characteristics including internal organ involvement and hand function were investigated in 160 consecutive patients with SSc (median age 46 (43;56) years; diffuse cutaneous SSc 55%).

**Results:**

A strong correlation was found between the ScleroID and articular disease activity scores (DAS28-CRP, DAS28-ESR, CDAI, SDAI), a hand function performance test, the Hand Anatomy Index and muscle strength tests. Additionally, a strong significant correlation was discovered using instruments representing hand function and musculoskeletal disability including the Cochin Hand Function Scale, the Quick Questionnaire of the Disability of the Hands, Arms and the Shoulders and the Health Assessment Questionnaire Disability Index. A significant negative correlation was found between the ScleroID score and the 6-min walking test (6MWT) (rho − 0.444, *p* < 0.001). Clinically mild lung/heart disease did not show increased ScleroID values. The Mouth Handicap in the Scleroderma Scale and the University of California Los Angeles Scleroderma Clinical Trials Consortium gastrointestinal tract 2.0 also showed significant positive correlations to the ScleroID score (rho: 0.626, *p* < 0.001; rho: 0.646, *p* < 0.001, respectively). Patients experiencing oesophageal difficulties bore a significantly higher score compared to individuals with a normal functioning oesophagus (3.2/1.5;4.5/ vs. 2.2/1.0;3.2/, *p* = 0.011). Moreover, the ScleroID showed a significant positive correlation to the revised EUSTAR disease activity index and modified activity index.

**Conclusion:**

In a large single-centre cohort, the previously described ScleroID-related findings were confirmed. Furthermore, several organ involvement-related functional and performance tests showed a good correlation to the ScleroID including the 6MWT and gastrointestinal-related complaints. Many aspects of musculoskeletal damage, overall disease activity, pain and fatigue were also well represented in the ScleroID, which efficiently reflects the impact of organ involvement, disease activity and functional damage.

## Introduction

Systemic sclerosis (SSc) is a multiorgan systemic disease affecting both internal organs and the musculoskeletal system resulting in decreased health-related quality of life (HRQoL) [1–3] and survival [4, 5]. Measuring HRQoL is challenging as since many aspects of quality of life are affected in SSc. It is well known that the general health in SSc (measured with Short-Form Health-Survey 36 (SF36)) is substantially impaired when compared to healthy subjects [6] and a high level of disability is present in patients affected with SSc [7–10].

HRQoL can be assessed using general instruments (e.g. SF-36) [11, 12], disease group instruments representing musculoskeletal disability (e.g. Health Assessment Questionnaire Disability Index (HAQ-DI)) [7, 11, 13] and disease-specific tools (e.g. University of California Los Angeles Scleroderma Clinical Trials Consortium gastrointestinal tract (UCLA-GIT) [14, 15]. A novel disease-specific questionnaire, a patient-reported outcome measure (PROM), was recently introduced for SSc, later named the EULAR Systemic sclerosis Impact of Disease (ScleroID). It was validated in a multicentre cohort [16], and recently it has been translated and validated in the Hungarian language [17]. Developing PROMs are essential tools used in combatting a complex disease such as SSc, since it aids in both the decision-making among physicians and patient satisfaction [18].

HRQoL in SSc is strongly influenced by internal organ involvement (e.g. cardiac or pulmonary involvement) resulting in fatigue and exertional dyspnoea [1, 3, 8, 9]. Patients’ gastrointestinal symptoms, severity of Raynaud’s phenomenon and fatigue, are the biggest contributors to decreased QoL [9, 19, 20]. HRQoL may deteriorate due to patients’ musculoskeletal and vascular symptoms, such as contractures, digital ulcers or joint swelling/pain [9, 19, 21–24]. Articular involvement can appear early in the course of the disease and may result in contractures and disability [10, 25, 26]. Measuring decreased hand function can be assessed through the administering of different tests, including the Hand Anatomy Index (HAI) [27] and the Hand Mobility in SSc scale (HAMIS) [28]. Additionally, questionnaires can be disseminated (e.g. the Cochin Hand Function Scale-CHFS, the Quick Questionnaire of the Disability of the Hands and the Arms and Shoulders-qDASH) [29–31].

The recently developed ScleroID PROM aimed to capture the patients’ evaluation regarding their disease severity. Following the prioritization of different aspects of health dimensions in SSc patients, ten different items were selected to constitute the questionnaire. The simple questionnaire showed a good correlation with patients’ global assessment and sHAQ, it has good re-test reliability and sensitivity to change and was designed to measure the overall disease impact [16]. Herein, we aimed to further investigate its clinical correlations with musculoskeletal and internal organ involvements in a large SSc cohort.

## Patients and methods

One hundred and sixty consecutive SSc patients fulfilling the 2013 ACR/EULAR criteria [32] were enrolled at the Department of Rheumatology and Immunology, Medical School, Pécs, Hungary, between June 2017 and July 2019. Patients with overlap syndromes were not excluded. The mean age of the patients was 55.7 ± 13.1 years. Median disease duration estimated from the first non-Raynaud symptom was 6.5 years (4;12). The disease duration in the subgroup of diffuse cutaneous SSc (dcSSc) was significantly lower when compared to the disease duration in the limited cutaneous SSc (lcSSc) subgroup (7.5/3;13/ vs. 12/7;17/, *p* = 0.001). Fifty-five per cent of patients (*n* = 88) had dcSSc. Fifty-five cases (34.4%) had a disease duration of less than 5 years, and among them 35 had dcSSc.

Internal organ involvement was defined based on the following criteria: Interstitial pulmonary involvement (ILD) was defined by two trained radiologists using high-resolution computed tomography (HRCT) [33]. Severe ILD was defined if the diffusing capacity of carbon monoxide (DLCO) was < 51% and forced vital capacity (FVC) < 80% with the presence of ILD on HRCT [34]. Pulmonary arterial hypertension (PAH) was recorded if > 25 mm Hg mean pulmonary arterial hypertension was detected by right heart catheterization (RHC) [35]. Patients having elevated right ventricular pressure on echocardiography were referred to RHC by the cardiologist based on a standard protocol. The overwhelming majority of the patients were examined by the same team of cardiologists [36]. Exertional dyspnea was a patient-reported item as reported during the interview. Heart disease was defined as the presence of either congestive heart disease, or pericardial disease or arrhythmia or relaxation disturbances [37]. The 6-min walk test (6MWT) was performed according to standard protocol [38] and the distance covered recorded. It was considered decreased in the case of less than 300 m [39]. Left ventricular mass index was also calculated based on echocardiographic results [36]. Oesophageal involvement was stated in the presence of stricture or dysmotility on barium swallow test [4]. GI involvement was categorized based on the UCLA GIT 2.0 score to mild, moderate and severe disease as described [14]. Scleroderma renal crisis was defined by a standard protocol [40]. HRCT findings were available in 134 cases, spirometry was performed in 159 cases, echocardiography was achieved in 146 cases and the 6 MWT was performed in 159 patients. Barium swallow test was performed in 153 patients, hand function tests were available in 159 cases and the set of questionnaires was duly reviewed and evaluated in all patients.

Disease activity was assessed using the European Scleroderma Study Group Activity Index (EScGAI), the Modified 9.5 point Activity Index developed by our team and also the revised EUSTAR Activity Index (EUSTAR-AI) [41–43]. Patients also completed the validated Hungarian version of the ScleroID tool [17].

To evaluate patients’ opinions, the validated Hungarian version of the self-assessment questionnaire was used including the HAQ-DI [13], CHFS [29], qDASH [30], skin thickness questionnaire [44], SF-36 [12], scleroderma HAQ (sHAQ) [45], Mouth Handicap in Scleroderma Scale (MHISS) [46] and UCLA-GIT 2.0 [14, 15]. The physician’s and patient’s global assessment, the joint pain and the degree of fatigue were also recorded through the use of a visual analogue scale (VAS).

To evaluate hand function in patients affected with SSc, validated articular activity scores including the Disease Activity Score 28 Erythrocyte Sedimentation Rate, the Disease Activity 28 Score C-Reactive Protein, the Simplified Disease Activity Index and the Clinical Disease Activity Index, (DAS28-ESR, DAS28-CRP, SDAI and CDAI, respectively) were calculated [24, 47]. Disability of the hand by performance tests was investigated with HAMIS [28] and HAI [27]. Muscle strength was described by calculating the Manual Muscle Test 8 (MMT8) [48]. Contractures were defined if the range of motion upon physical examination was less than 75% [22].

### Ethics

All patients submitted their informed written consent to the study, which was conducted in full accordance with the Declaration of Helsinki and was approved by the National Research Ethics Committee (30,636–3/2017/EKU).

### Statistical analysis

SSc subgroups were compared using the Mann–Whitney *U* or the Kruskal–Wallis test in the case of continuous variables. Correlation analysis was performed using the Spearman correlation analysis. Since most of the continuous variables showed no normal distribution, data are given as median (lower quartile; upper quartile). The Random Forest (RF) analysis was used to detect potential predictors of the 6MWT, in which the significant contributors were placed into linear regression analyses to determine the independent contributors of 6MWT. Data were analysed using Statistica 6.0 (Tulsa, USA).

## Results

Clinical characteristics are detailed in Table 1.

**Table 1:** Clinical characteristics of 160 systemic sclerosis patients

	*N* = 160 (%)
Female	138 (86.3)
Diffuse cutaneous SSc	88 [55]
Modified Rodnan skin score (median (IQR)	6.5 (4;12)
Modified Rodnan skin score (median (IQR) in diffuse cutaneous SSc	10 (6;15)
Modified Rodnan skin score (median (IQR) in limited cutaneous SSc	4 (2;7)
Presence of Raynaud’s phenomenon	154 (96.3)
Current digital ulcers	12 (7.5)
Digital ulcer ever (including current)	52 (32.5)
Presence of current synovitis	18 (11.3)
Joint contractures	91 (56.9)
New York Heart Association cardiac state III–IV	13 (8.1)
Exertional dyspnoea	100 (62.5)
Lung fibrosis detected by high-resolution computer tomography	97 (60.6)
Cardiac involvement	93 (58.1)
Pulmonary arterial hypertension by right heart catheterization	5 (3.1)^a^
Scleroderma renal crisis	1 (0.6)
Mild gastrointestinal involvement by UCLA GIT 2.0	123 (76.9)
Moderate gastrointestinal involvement by UCLA GIT 2.0	22 (13.8)
Severe gastrointestinal involvement by UCLA GIT 2.0	15 (9,4)
Malabsorption	21 (13.1)
Gastrointestinal pseudo-obstruction	3 (1.9)
Use of cyclic antibiotics for gastrointestinal dysmotility	18 (11.3)
Gastric vascular ectasia	3 (1.9)
Anti-centromere	35 (21.9)
Anti-topoisomerase I	39 (24.4)
Anti-RNA polymerase III	22 (13.8)
Anti-PmScl	16 (10)
Anti-Ku	6 (3.8)
Anti-Th/To	7 (4.4)
Anti-Nor90	4 (2.5)
Anti-U3RP	3 (1.9)
No scleroderma-specific antibody	55 (34.4)
Rheumatoid factor	53 (33.1)
Anti-cylic citrullinated peptide	10 (6.3)

^a^Based on a standard protocol [34] 14 patients were referred to right heart catheterization by our cardiologist team

The median overall ScleroID score was 2.3 (1.05;3.65). Regarding the SSc subsets, dcSSc cases had scored 2.3 (0.95;3.65) versus 2.4 (1.25;3.65) for lcSSc patients. There was no significant difference between the two subgroups (*p* = 0.856). The ScleroID score showed a good correlation with the Patient’s Global Assessment, yet not with the Physician’s Global Assessment in both lcSSc and dcSSc subsets. Furthermore, significant correlations were found with SF-36, HAQ-DI and SHAQ in both subgroups (Table 2).

**Table 2:** Correlation analysis of different self-assessment questionnaires and the ScleroID

Variable	SSc (*n* = 160)	dcSSc (*n* = 88)	lcSSc (*n* = 72)
	rho (*p*)	rho (*p*)	rho (*p*)
Physician’s Global Assessment	0.12 (0.105)	0.07 (0.471)	0.19 (0.113)
Patient’s Global Assessment	0.53 (< 0.001)	0.51(< 0.001)	0.55 (< 0.001)
SF-36 Physical component score	− 0.75 (< 0.001)	− 0.81 (< 0.001)	− 0.68 (< 0.001)
SF-36 Mental component score	− 0.62 (< 0.001)	− 0.66 (< 0.001)	− 0.57 (< 0.001)
HAQ-DI	0.64 (< 0.001)	0.68 (< 0.001)	0.60 (< 0.001)
SSc-HAQ	0.62 (< 0.001)	0.68 (< 0.001)	0.56 (< 0.001)
VAS-GIT	0.51 (< 0.001)	0.48 (< 0.001)	0.55 (< 0.001)
VAS-Dyspnea	0.50 (< 0.001)	0.46 (< 0.001)	0.55 (< 0.001)
VAS-Raynaud	0.58 (< 0.001)	0.61 (< 0.001)	0.55 (< 0.001)
VAS-Ulcers	0.36 (< 0.001)	0.41 (< 0.001)	0.30 (0.01)

*SF-36* Short-Form Health-Survey 36, *HAQ-DI* Health Assessment Questionnaire Disability Index, *VAS* Visual analogue scale, *SSc-HAQ* Scleroderma HAQ

The SF-36 Mental Component Score (SF-36 MCS) showed no significant difference between lcSSc and dcSSc (73.0/46.1;85.4/ vs. 59.1/37.6;81.0/; *p* = 0.062), while the SF-36 Physical Component Score (SF.36 PCS) was significantly higher in dcSSc (43.7/24.7;64,2/ vs. 51.9/28.7;76.2/; *p* = 0.038). When early and late disease in both subsets were compared, no significant difference was found regarding SF-36 MCS nor SF-36PCS. No difference was observed in consideration of HAQ-DI between lcSSc and dcSSc patients (data not shown). Patients with HAQ-DI ≤ 1 had significantly lower ScleroID scores when compared to the counterparts with HAQ-DI > 1 (1.4/0.7;2.4/ vs. 3.5/2.5;4.8/, *p* < 0.001).

Patients suffering from cardiac involvement did not have a significantly higher ScleroID score compared to those without (2.5/1.4;3.7/ vs. 2/0.9;3.5/, *p* = 0.173). No significant difference was found regarding the ScleroID score between patients with a preserved ejection fraction (> 50%) and a decreased ejection fraction (< 50%), between cases with and without diastolic dysfunction on echocardiography, and, with and without conduction disturbances on electrocardiogram (data not shown).

**Fig. 1:**
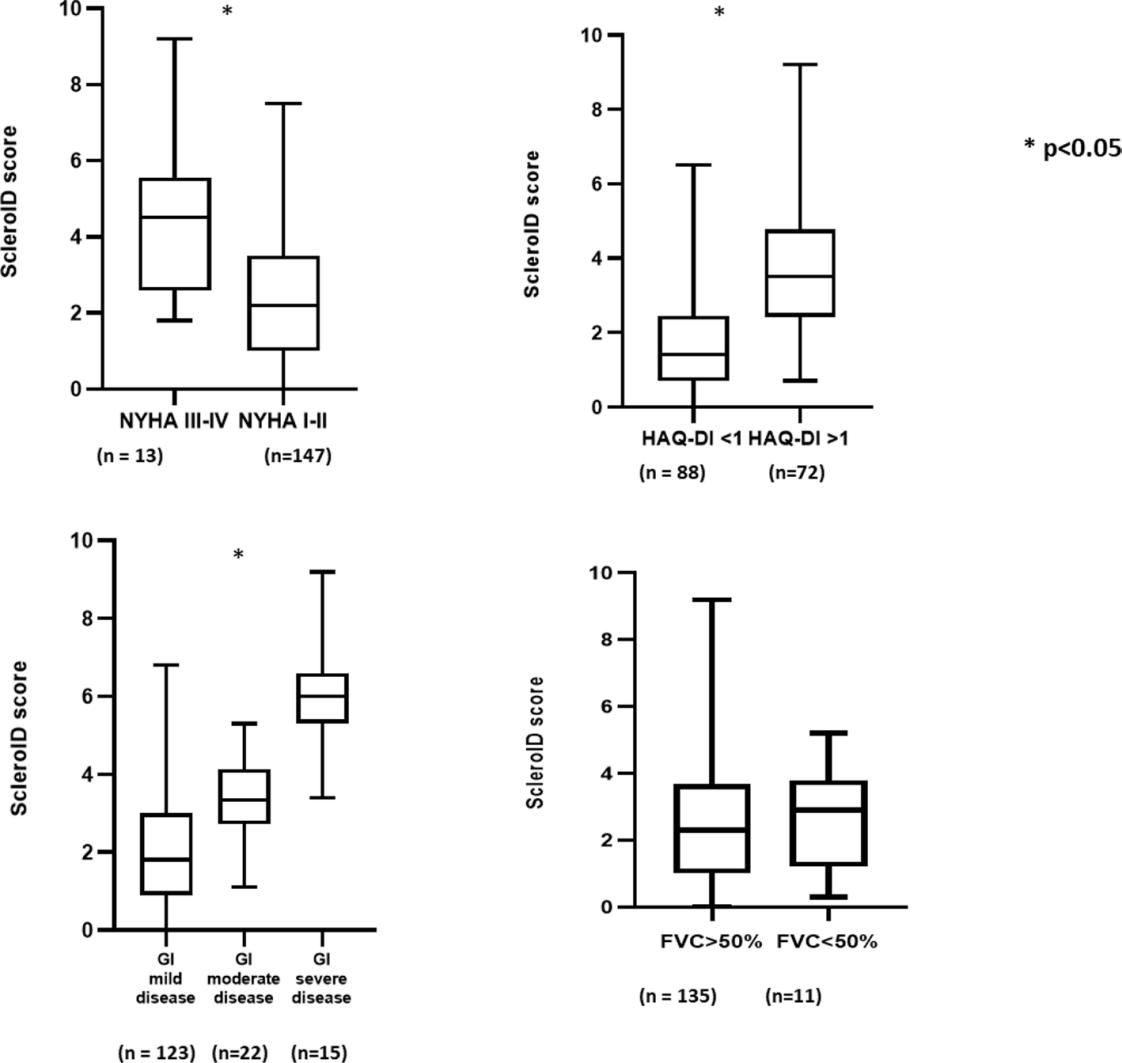
Comparison of ScleroID values of cases with mild versus severe organ involvement. NYHA, New York Heart Association; HAQ-DI, Health Assessment Questionnaire disability Index; GI, gastrointestinal, ScleroID, EULAR Systemic sclerosis Impact of Disease; FVC, forced vital capacity

The ScleroID score showed a significant moderate negative correlation with the distance performed in the 6MWT (rho: − 0.440, *p* < 0.001), but not with calculated right ventricular pressure (cRVP) (rho: 0.051, *p* = 0.641) (Table 3). Patients with a decreased distance covered in the 6MWT had significantly lower ScleroID scores when compared to those with normal distances covered (1.95/1.0;3.3/ vs, 3.9/3.0;5.3/, *p* < 0.001). Patients with dyspnea class NYHA III/IV had significantly higher ScleroID scores when compared to patients with NYHA class I/ II (4.5/2.8;5.4/ vs. 2.2/1.0;3.5/, *p* < 0.001) (Fig. 1).

**Table 3:** Clinical correlations of ScleroID scores^a^

	All SSc cases (*n* = 160)		DcSSc (*n* = 88)		lcSSc (*n* = 72)	
	Rho	*p*	Rho	*p*	Rho	*p*
Number of current digital ulcers on hands (currently present in 12 patients)	0.095	0.236	0.069	0.526	0.135	0.257
Number of shortened digits (present in 35 patients)	0.180	0.023	0.266	0.012	0.038	0.750
Number of digital pitting scars (currently present in 57 patients)	0.180	0.026	0.197	0.072	0.163	0.182
DAS28 ESR	0.358	< 0.001	0.418	< 0.001	0.258	0.029
DAS28CRP	0.441	< 0.001	0.480	< 0.001	0.373	< 0.001
SDAI	0.484	< 0.001	0.508	< 0.001	0.426	< 0.001
CDAI	0.488	< 0.001	0.518	< 0.001	0.428	< 0.001
Tender joint	0.332	< 0.001	0.436	< 0.001	0.193	0.104
Swollen joint	0.173	0.029	0.160	0.139	0.182	0.126
Modified Rodnan skin score	-0,194	0.015	-0,236	0.028	-0,229	0.055
EULAR revised activity score	0.256	0.001	0.174	0.103	0.379	0.001
Modified activity score	0.476	< 0.001	0.502	< 0.001	0.453	< 0.001
Calculated right ventricular pressure by echocardiography	0,051	0.641	-0,072	0.522	0.160	0.210
6-min walk distance	-0,440	< 0.001	-0,433	< 0.001	-0.443	< 0.001
HAMIS right	0.331	< 0.001	0.455	< 0.001	0.163	0.170
HAMIS left	0.282	< 0.001	0.386	< 0.001	0.160	0.180
Hand Anatomy Index Right	-0.283	< 0.001	-0.350	< 0.001	-0.175	0.140
Hand Anatomy Index Left	-0.327	< 0.001	-0.427	< 0.001	-0.183	0.122
Number of contractures	0.185	0.020	0.260	0.015	0.090	0.449
Manual Muscle Test 8	-0.457	< 0.001	-0.602	< 0.001	-0.331	0.005
Mouth Handicap in Scleroderma Scale	0.626	< 0.001	0.568	< 0.001	0.678	< 0.001
Skin thickness questionnaire	0.248	0.002	0.215	0.045	0.325	0.005
Cochin Hand Function Scale	0.710	< 0.001	0.673	< 0.001	0.753	< 0.001
qDASH	0.765	< 0.001	0.797	< 0.001	0.718	< 0.001
UCLA-GIT	0.646	< 0.001	0.567	< 0.001	0.740	< 0.001
VAS pain scale	0.652	< 0.001	0.624	< 0.001	0.673	< 0.001
VAS joint paint	0.573	< 0.001	0.578	< 0.001	0.539	< 0.001
VAS fatigue scale	0.651	< 0.001	0.729	< 0.001	0.555	< 0.001

*DAS28 ESR* Disease Activity Score 28 Erythrocyte Sedimentation Rate, *DAS28 CRP* Disease Activity 28 Score C-Reactive Protein, *SDAI* Simplified Disease Activity Index, *CDAI* Clinical Disease Activity Index, *EULAR*, *HAMIS* Hand Mobility in SSc scale, *qDASH* Quick Questionnaire of the Disability of the Hands, Arms and Shoulders, *UCLA-GIT* University of California Los Angeles Scleroderma Clinical Trials Consortium gastrointestinal tract 2.0, *VAS* Visual analogue scale

^a^Regarding missing values, different measurements were available in at least 157 cases except for echocardiography, in which the results of a recent investigation were available in 84 cases

Based on binary logistic regression modelling, age, left ventricular mass index and the MMT8 score were the significant contributors to the distance performed on 6MWT (data not shown). When patients with knee contractures (*n* = 4) and patients with decreased MMT8 (*n* = 15) were excluded, the correlation between the ScleroID score and the 6MWT still remained significant (rho − 0.456 *p* < 0.001, rho − 0.381 *p* < 0.001, respectively).

Neither patients having ILD compared to cases without (2.5/1.2;4.0/ vs. 2.2/1.0;3.5/, *p* = 0.212), nor the patients having severe ILD compared to patients without (2.8/1.5;4.5/ vs. 2.3/1.0;3.6/ *p* = 0.547) showed significant differences in the ScleroID score. Patients with ≤ 50% FVC (*n* = 11) showed no significant difference regarding ScleroID score when compared to those with FVC > 50% (*n* = 148) (2.9 /1.2;3.8/ vs 2.3 /1.0;3.7/ *p* = 0.817).

Comparison of patients’ UCLA GIT 2.0 values showed a significant difference, in which patients with a mild case of the disease had the lower and the patients with severe disease had the highest ScleroID scores (1.8/0.9;3.0/ vs. 3.4/2.8;4.1/ vs. 6.0/5.3;6.6/, *p* < 0.001). Patients with oesophageal involvement had significantly higher scores when compared to those without (3.2/1.5;4.5/ vs. 2.2/1.0;3.2/, *p* = 0.011). Patients having malabsorption also had significantly higher ScleroID scores when compared to those without (3.5/2.3;3.9/ vs. 2.2/1.0;3.6/, *p* = 0.010) (Fig. 1).

Additionally, the ScleroID score showed strong correlations with the revised EULAR Activity Index (rho: 0.256, *p* = 0.001) and with the modified activity index (rho: 0.476, *p* < 0.001).

**Fig. 2:**
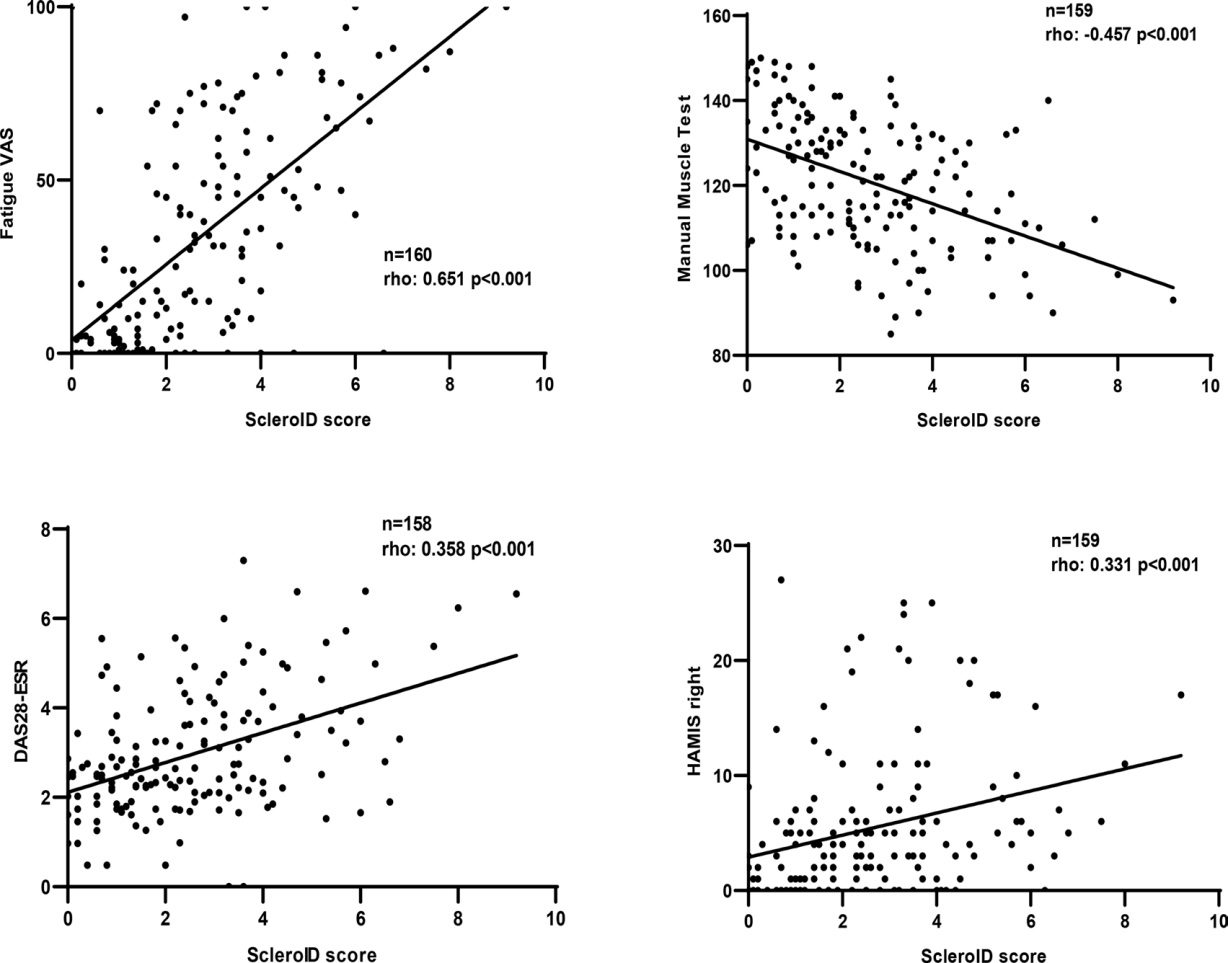
Clinical correlations of ScleroID and musculoskeletal performance tests, fatigue and DAS28-ESR. ScleroID, EULAR Systemic sclerosis Impact of Disease; VAS, visual analogue scale; DAS28, disease activity score erythrocyte sedimentation rate; HAMIS, hand mobility in systemic sclerosis scale

The ScleroID score showed a strong significant correlation with all articular disease activity scores (DAS28-CRP, DAS28-ESR, CDAI and SDAI) in the entire cohort and in both SSc subtypes. However, the number of swollen joints showed significant correlations with the ScleroID only upon examination of the entire cohort The ScleroID score also showed a significant positive correlation with hand disability indices including HAMIS in both hands and the number of contractures in the entire cohort (rho: 0.331, *p* = 0.000; rho: 0.185, *p* = 0.020, respectively). Regarding HAI, a significant negative correlation was found with the ScleroID in both hands (rho: − 0.283, *p* < 0.0001). Muscle strength evaluated by MMT-8 showed a significantly strong correlation with the ScleroID in all investigated groups (Fig. 2).

Patients with current or previous digital ulcers (DU) had significantly higher ScleroID scores when compared to those without DUs (3.2/1.6;4.0/ vs. 2.1/0.9;3.2/, *p* = 0.005); however, the ScleroID score did not correlate with the current number of DU. The calculated ScleroID score showed significant correlations with the number of shortened digits also (entire cohort rho: 0.18, *p* = 0.022; dcSSc: rho: 0.27 *p* = 0.010).

The analysis regarding self-assessment questionnaires showed strong correlations with all investigated questionnaires (MHISS, Skin thickness questionnaire, CHFS, qDASH and UCLA-GIT) in the entire cohort and both subsets. Lastly, both pain and fatigue visual analogue scales showed significant positive associations with the ScleroID score.

## Discussion

SSc is a complex disease, therefore it is difficult to characterize patients with low HRQoL, since the factors negatively affecting patients’ HRQoL are highly variable. Several tools are available to examine HRQoL in SSc patients [12, 13], yet only a few instruments are specific for SSc [14, 15, 45, 46]. Physicians predominantly focus on severe organ complications; however, these may not always be the main contributor to deteriorating HRQoL [1, 3, 8, 9, 19, 20]. The newly introduced ScleroID tool was developed with the help and contribution of SSc patients and covers the most important health dimensions which may affect HRQoL among SSc patients. First, we confirmed the associations identified by Becker and Dobrota et al. in our single-centre large SSc cohort. The Hungarian-validated version of ScleroID demonstrated a good performance. In our hands, the ScleroID tool showed a significant correlation with the Patient’s Global Assessment, yet not with Physician’s Global Assessment. This parallels previous observations which showed patients’ and physicians’ experiences regarding disease severity indeed differ [16, 49]. In the original ScleroID validation paper, Raynaud`s phenomenon, impaired hand function, pain and fatigue had the highest patient-reported disease impact evaluated by VAS [16]. In our single-centre study, we confirmed the importance of these particular findings (Table 3). Our single-centre study also confirmed good correlations identified by Becker and Dobrota et al. between widely used questionnaires (SF-36, HAQ-DI and sHAQ) and the ScleroID, allowing us to investigate further correlations [16] (Table 2). The lack of significant differences in ScleroID scores between dcSSc and lcSSc subsets may be partially explained by the high prevalence of musculoskeletal and GI involvements in both subsets and cardio-pulmonary manifestations also showed high prevalence in both subsets. Furthermore, dcSSc patients have a worse survival, and early severe cases are not enrolled to our cohort due to their early demise (in our previous paper, 19 out 106 deceased patients succumbed in the first 2 years to the disease) [4, 50, 51].

In our study, several functional and performance tests showed a moderate to good correlation regarding the ScleroID. These included the 6MWT reflecting overall cardiorespiratory function and GI-related complaints represented by MHISS and UCLA-GIT 2.0. Furthermore, both inflammatory joint symptoms and articular damage reflected by DAS28, CDAI, SDAI and qDASH scores and the overall hand disability tests correlated with the ScleroID. Muscular function measured by MMT8 is also well correlated with the ScleroID. Notably, a good correlation with overall disease activity was found. Additionally, important items including overall and articular pain and fatigue were also well represented in ScleroID. Conversely, the presence of internal organ involvements and digital ulcers, number of joint contractures and skin score did not substantially influence the ScleroID. Identification of the presence of a certain organ manifestation does not provide sufficient information in reference to its contribution to the decreased HRQoL of SSc patients.

Regarding the impaired hand function, we have shown both arthritis and irreversible joint damage are important contributors to the impaired ScleroID. Not surprisingly, significant correlations were identified with different measurements evaluating hand function. Hand function was implemented into the tool; it was the second most important parameter in the Delphi exercise indicating it distinctively impacts patients’ well-being [47]. Both activity scores (DAS28-CRP, DAS28-ESR, CDAI, SDAI) evaluating articular activity and performance test and quantitative measurements (HAMIS, HAI) assessing damage along with the number of contractures showed strong correlations with ScleroID score, though the latest not in lcSSc. It can be explained by the fact articular damage is more pronounced in dcSSc. Calculating the ScleroID score may help identifying patients with impaired hand function, especially in dcSSc. Joint tenderness, but not swelling, also showed a strong correlation in dcSSc. It is aligned with previous observations, in which tenderness, but not swelling, is more prevalent in SSc [52]. Seemingly, the new tool better represents articular pain than current inflammatory changes.

In addition to hand function, other important associations regarding the ScleroID score included the number of shortened digits, yet not the number of current DU. This may be explained due to the low prevalence of patients with current DU, which was less than in the cohort previously published by Becker et al. (13% vs. 7.5%). Presence of DU was also implemented in the tool [16].

Regarding internal organ involvements, ScleroID score showed a significant negative correlation with 6MWT. Decreased 6MWT may result from various causes including ILD, PAH cardiac or musculoskeletal involvement, and its decreased value is associated with a poor outcome [53]. From Sanges et al., the 6 MWT reflects more pulmonary vasculopathy than lung parenchymal involvement in SSc [54] and shows independent associations with the HAQ-DI. It is important to highlight, the ScleroID appropriately reflects the 6MWT. Seemingly, it is a valuable, simple and feasible instrument during follow-up among patients. The ScleroID score of patients affected with NYHA III-IV was also significantly impaired compared to the remainder of the cases. Regarding lung and heart involvement, the ScleroID was not sensitive enough for clinically mild symptoms; otherwise, it suitably reflected severe cardiorespiratory symptoms as patients with NYHAIII/IV state had significantly lower scores on ScleroID.

Significant associations were identified between the ScleroID tool and one of the most important contributors of decreased HRQoL, gastrointestinal involvement [19, 20] investigated by both objective and subjective tools. The newly introduced tool superbly reflects gastrointestinal involvement, especially its severe form, and the objectively proven oesophageal involvement (dysmotility or stricture on barium swallow test). Furthermore, the ScleroID showed correlations with two different self-assessment questionnaires investigating GI involvement, specifically, the MHISS and the UCLA-GIT 2.0 [14, 15, 46]. The new tool not only represented both major GI manifestations, oesophageal and bowel disease, but also correlated well with the symptoms regarding oral manifestations of the disease. Hence, the new tool may positively impact patients’ well-being along the entire GI tract.

The EUSTAR revised AI showed a good correlation with the ScleroID in all investigated subgroups [41–43]. The ScleroID may prove helpful in identifying patients with high disease activity along with other previously established factors. AI might be assessed in patients with long disease duration; however, the recently introduced composite response index in dcSSc due to the relatively low number of early dcSSc cases in this particular cross-sectional study was not assessed [55]. Previously some organ-specific questionnaires were developed to evaluate organ involvement in SSc patients [14, 29, 30, 46]. CHFS and qDASH showed associations with hand arthritis and flexion contractures, therefore, were able to detect patients with impaired hand function [22, 24, 26]. All SSc-specific questionnaires showed good correlations with the ScleroID and may prove beneficial to complement the use of the previously developed tools.

Our data suggest the ScleroID reflects both activity and damage associated with SSc. The items implemented to the tool covered the majority of different health dimensions playing a role in deteriorating HRQoL.

Other significant contributors regarding the decreased HRQoL [3] including fatigue and pain also showed significant strong correlations with the newly introduced tool, not surprisingly, since both items were implemented to the tool. This is aligned with the results of the Delphi exercise preceding the development of the tool: Patients ranked pain and fatigue as the fourth and fifth most important characteristic [16]. Fatigue is associated with many aspects of SSc, including GI symptoms, articular involvement and breathlessness [56], all showing strong correlations with the investigated tool, confirming its usefulness.

Our study bears several limitations. The ScleroID was designed as an overall measure of disease impact. The fact the score calculated in lcSSc and dcSSc showed no significant difference may be explained with no prior selection criteria established. Patients with both short and long-standing diseases were enrolled from both subsets, and patients with long disease duration may have developed coping mechanisms, while patients with early disease may have no severe complications. In our current cohort, disease duration was significantly longer in lcSSc patients when compared to dcSSc patients. Further analysis of data originating from different centres is essential to substantiate the real impact of the tool.

## Conclusion

In conclusion, the validated Hungarian version of ScleroID performed well and all the basic findings described in the initial paper were effectively reproduced. The ScleroID reflects the GI manifestations of the disease, the deteriorated hand function, pain and fatigue, all surprisingly well. On the other hand, its performance in identifying patients with cardiopulmonary organ involvement was poor, since it is not sensitive enough to select patients with clinically mild involvement. However, the ScleroID well represented severe heart/pulmonary involvement measured by 6MWT and NYHA stage. The ScleroID covers many aspects of HRQoL, therefore, this particular newly developed instrument may help to identify patients with decreased HRQoL caused by both musculoskeletal and severe organ involvement.

## Data Availability

The datasets generated and/or analysed during the current study are not publicly available due to ethical issues but are available from the corresponding author on reasonable request.

## Abbreviations

CDAI: Clinical Disease Activity Index
CHFS: Cochin Hand Function Scale
cRVP: Calculated right ventricular pressure
DAS28-CRP: Disease Activity 28 Score C-Reactive Protein
DAS28-ESR: Disease Activity Score 28 Erythrocyte Sedimentation Rate
dcSSc: Diffuse cutaneous SSc
DLCO: Diffusing capacity of carbon monoxide
EScGAI: European Scleroderma Study Group Activity Index
EUSTAR-AI: Revised EUSTAR Activity Index
FVC: Forced vital capacity
HAI: Hand Anatomy Index
HAMIS: Hand Mobility in SSc scale
HAQ-DI: Health Assessment Questionnaire Disability Index
HRCT: High-resolution computed tomography
HRQoL: Health-related quality of life
ILD: Interstitial pulmonary involvement
lcSSc: Limited cutaneous SSc
MHISS: Mouth Handicap in Scleroderma Scale
MMT8: Manual Muscle Test 8
PAH: Pulmonary arterial hypertension
PROM: Patient-reported outcome measure
qDASH: Quick Questionnaire of the Disability of the Hands and the Arms and Shoulders
RF: Random Forest
RHC: Right heart catheterization
ScleroID: EULAR SSc Impact of Disease
SDAI: Simplified Disease Activity Index
SF36: Short-Form Health-Survey 36
SF-36 MCS: SF-36 Mental Component Score
SF.36 PCS: SF-36 Physical Component Score
sHAQ: Scleroderma HAQ
SSc: Systemic sclerosis
UCLA-GIT: University of California Los Angeles Scleroderma Clinical Trials Consortium gastrointestinal tract 2.0
VAS: Visual analogue scales
6MWT: Six-minute walking test
